# Atomic Layer Deposition as the Enabler for the Metastable Semiconductor InN and Its Alloys

**DOI:** 10.1021/acs.cgd.3c00775

**Published:** 2023-09-19

**Authors:** Henrik Pedersen, Chih-Wei Hsu, Neeraj Nepal, Jeffrey M. Woodward, Charles R. Eddy

**Affiliations:** † Department of Physics, Chemistry and Biology, Linköping University, SE-581 83 Linköping, Sweden; ‡ Electronics Science and Technology Division, U.S. Naval Research Laboratory, Washington, District of Columbia 20375, United States

## Abstract

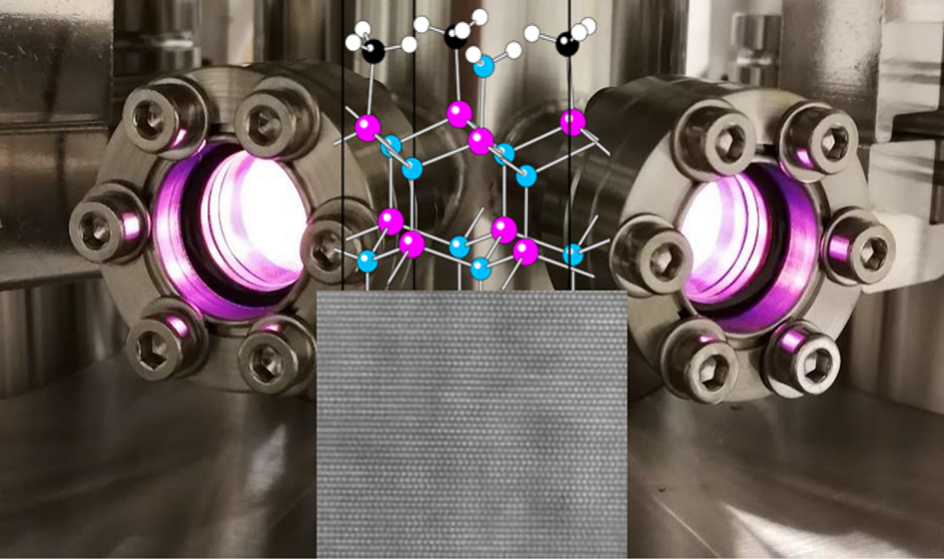

Indium nitride (InN) is a low-band-gap semiconductor with unusually high electron mobility, making it suitable for IR-range optoelectronics and high-frequency transistors. However, the development of InN-based electronics is hampered by the metastable nature of InN. The decomposition temperature of InN is lower than the required growth temperature for most crystal growth techniques. Here, we discuss growth of InN films and epitaxial layers by atomic layer deposition (ALD), a growth technique based on self-limiting surface chemical reactions and, thus, inherently a low-temperature technique. We describe the current state of the art in ALD of InN and InN-based ternary alloys with GaN and AlN, and we contrast this to other growth technologies for these materials. We believe that ALD will be the enabling technology for realizing the promise of InN-based electronics.

## Abstract 2

Indium nitride (InN) is a low-band-gap semiconductor with unusually high electron mobility, making it suitable for IR-range optoelectronics and high-frequency transistors. This potential is hampered by the breakdown temperature of InN, which is lower than the required growth temperature for most crystal growth techniques. We describe the current state of the art in atomic layer deposition (ALD) of InN and InN-based ternary alloys with GaN and AlN and argue that ALD will be the technology for realizing the promises of InN-based electronics.

## Introduction

Indium nitride (InN) forms, together with aluminum nitride (AlN) and gallium nitride (GaN), the family of semiconductors commonly known as the group III nitrides or III-Ns for short. The III-Ns hold great technological promise and are already today key materials for several electronic applications, such as light emitting diodes. The revised band gap of InN from 1.9 eV down to 0.7 eV^1^ and a predicted room temperature low-field electron mobility of up to 14000 cm^2^/(V s)^2^ make InN a promising material for optoelectronics and high-frequency applications, such as IR emitters,^3^ sensors,^4^ photovoltaics,^5^ thin-film transistors,^6^ and high-electron-mobility transistors.^7^ The utilization of InN and high-In-content InN-based alloys is the key to extend these applications to longer wavelength and higher operation frequency regimes. The III-Ns can be alloyed with each other since they all, predominantly, crystallize in the wurtzite structure. This allows, at least in theory, for the formation of ternary nitrides, i.e., Al_1–*x*_Ga_*x*_N, In_1–*x*_Ga_*x*_N, and In_1–*x*_Al_*x*_N with tunable bandgaps, spanning from the UV-range, with 6.2 eV for AlN, via the whole visible range with 3.4 eV for GaN, to the IR-range, with the 0.7 eV for InN.

One example where III–N technology has made a major impact is the modern light-emitting diode (LED). Most modern LEDs largely rely on the stacking of In_1–*x*_Ga_*x*_N/GaN multilayers as optically active layers, with the control of both thickness and composition in the In_1–*x*_Ga_*x*_N layers as the key to a high-quality LED. The efficiency of such LEDs decreases drastically with increasing emission wavelength, which has a direct correlation with the In content. Such an efficiency droop can be ascribed to phase separation as well as strain relaxation in the In_1–*x*_Ga_*x*_N layer caused by high In content.^8^ Achieving high-quality In_1–*x*_Ga_*x*_N with high In content is essential for further development of nitride-based LEDs toward longer emission wavelengths in the red part of the visible spectrum.

Another example is that In_1–*x*_Al_*x*_N plays an essential role in the evolution of nitride-based high-electron-mobility transistors. In_1–*x*_Al_*x*_N with a composition tuned to be lattice-matched to the underlying GaN is suggested to be better than using the current standard Al_1–*x*_Ga_*x*_N in GaN-based high-electron-mobility transistors (HEMTs). The In_1–*x*_Al_*x*_N allows a thinner barrier and avoids strain-related degradation, enabling more stable power output at higher operation frequency.^9^ Furthermore, high-In-content In_1–*x*_Al_*x*_N in combination with InN is proposed for next-generation HEMTs, which have the potential to extend the operation regime.^7^ Analogous to the LED application, achieving high-quality In_1–*x*_Al_*x*_N with high In content as well as thin high-quality InN is the solution to this next-generation HEMT.

The InN can stabilize in wurtzite (*P*6_3_*mc*), zinc-blende (*F*43*m*), and rock-salt (NaCl, B1) structures.^10^ The most thermodynamically stable phase of InN is the hexagonal wurtzite structure. Relatively low growth temperature and/or growth surface reactivity modifications are required to grow metastable phases. Oseki et al. reported cubic InN(111) films on yttria stabilized zirconia (111) substrates and demonstrated field effect transistors using high-quality ultrathin cubic InN channel layers by the pulsed-sputtering deposition technique.^11^ Both low temperatures and growth surface chemistry help to stabilize metastable phases.

In the proposed device structures for all applications of InN and InN alloys, the active regions are typically based on nanometer-thin, homogeneous layers, epitaxially grown between hosting matrix materials. Despite the attractive potential, growth of such material structures remains challenging, according to the tremendous research efforts made globally. The problem can be ascribed to the weak In–N bond, which is weaker than both the In–In and N–N bonds, making the InN crystal unstable with respect to In metal and N_2_ gas.^12^ This is manifested as precipitation of In nanoparticles separating out from InN and even formation of metallic In droplets on the surface. The equilibrium N_2_ pressure over InN is also much higher than the other III-Ns, GaN and AlN, leading to InN decomposition at the relatively low temperature of 630 °C, compared to 850 and 1040 °C for GaN and AlN, respectively.^13^ Some studies report InN decompositions at even lower temperatures.^14^ The inherent large lattice constant of InN is also an issue when growing it on foreign substrates, leading to a critical thickness before the onset of dislocations of InN on GaN of only 1 monolayer.^15^ The relaxation by formation of dislocations and thus high density of structural defects is almost inevitable in InN-based heterostructures.

Strategically, such issues can be tackled by applying the same concept as has been used in arsenide material systems—growing InN on In_1–*x*_Ga_*x*_N and In_1–*x*_Al_*x*_N to minimize the lattice mismatch. However, homogeneous In_1–*x*_Ga_*x*_N and In_1–*x*_Al_*x*_N with high In content are even more difficult to obtain due to their miscibility gaps. The predicted temperatures of forming homogeneous In_1–*x*_Ga_*x*_N and In_1–*x*_Al_*x*_N (known as critical temperature) are higher than their typical growth temperature,^16^ meaning that phase separation is thermodynamically favored especially for In-rich cases. For the most studied In_1–*x*_Ga_*x*_N, phase separation tends to occur for In content >20%,^17^ even in LED structures where the thickness of the In_1–*x*_Ga_*x*_N is below 5 nm.^18^

The opportunities and challenges described above make InN, In_1–*x*_Ga_*x*_N, and In_1–*x*_Al_*x*_N some of the most promising, and at the same time challenging, semiconductor materials today. Their challenges lie mainly in their crystal growth as homogeneous and epitaxial layers. This perspective summarizes our work on growing InN and InN alloys by atomic layer deposition (ALD). Since ALD relies on sequential and self-limiting formations of molecular monolayers on a surface (see below), ALD is limited to lower temperatures. It was therefore hypothesized that ALD could be a way forward for growing InN and InN alloys. We will describe results obtained from ALD of InN and InN alloys demonstrating epitaxial growth on foreign substrates without any signs of In droplet formation or phase separation. As we describe below, we believe that the ALD technique is an enabler for InN thin films, capable of realizing new devices relying on the high electron mobility in InN, e.g., high-frequency transistors.

## Brief Overview of Thin Film Deposition Techniques for InN

Thin film growth of electronic materials, especially semiconductors, has been predominantly achieved by three methods: metalorganic chemical vapor deposition (MOCVD), molecular beam epitaxy (MBE), and sputtering. Each of these has advantages and disadvantages in general and specifically in growing InN-based semiconductors which will be briefly highlighted in the following paragraphs.

MOCVD is a thermodynamically stable growth process that involves flowing a mixture of gases over a heated substrate, where the growth occurs. The process is well established in both the research and development and manufacturing communities and has been widely used in the manufacture of most semiconductor devices.^19^ In this process, the precursors for film growth are introduced into the gas stream before reaching the heated growth zone and are carried by relatively inert or nonreactive gases such as hydrogen or argon. For the group III element of the semiconductor (indium for InN, for example), the precursor is a metal–organic molecule (trimethylindium, for example) that is derived from a liquid or solid source through evaporation or sublimation into the carrier gas stream. For the group V element, the precursor is a gas or vapor (ammonia, for example). To avoid any prereaction and resulting particle formation, these streams are mixed just prior to being directed into the heated growth zone, often with careful attention to flow dynamics to improve uniformity of growth rate and film quality, where upon entering the hot zone the precursors become activated to promote chemical reactions—preferably on the heated substrate surface—that lead to growth of the semiconductor. Key parameters in the process are the temperature of the growth zone, the pressure in the growth reactor, and the ratio of group V to group III atoms in the gas phase. The quality of the resulting films—both structural and electronic—depends on these parameters as well as the quality of the substrate bulk and surface, the substrate’s suitability for lattice-matched growth, purity of precursors, and the overall contamination level of the growth system. For ternary and quaternary films these trade-offs are even more constrained by the spinodal and binodal decompositions (driven by temperature and strain) leading to local variations in alloy compositions.

MOCVD of InN and related materials has traditionally been performed using trimethylindium or triethlyindium and ammonia precursors but with more inert carrier gases (such as nitrogen) than hydrogen in the temperature range from 550 to 800 °C and at pressures between 75 and 500 Torr on sapphire or silicon substrates.^20,21^ The use of nitrogen instead of hydrogen has been shown to improve growth rates due to the reduced reaction of hydrogen with indium on the surface, resulting in volatile indium hydride products. However, the low growth temperatures, necessitated by the low dissociation temperature of InN and high desorption rate of indium, present a challenge for growth rates by MOCVD as they present low ammonia dissociation rates. This can be overcome to a degree either through the use of higher CVD pressures^22^ or plasma discharges (plasma-assisted or remote-plasma CVD)^23,24^ to dissociate the nitrogen precursor by nonthermal means, but this brings additional parameters into the growth process and can lead to increased contamination in resulting films. An alternative but closely related process—hydride vapor phase epitaxy (HVPE)—has more recently been used to grow InN films at high growth rates (12.4 μm/h) with some success.^25^

The other most prominent thin film growth method for semiconductor materials is MBE.^26^ MBE is a physical vapor deposition process where, traditionally, the precursor elements are evaporated from a pure solid or liquid source under high or ultrahigh vacuum conditions. These separate fluxes of atoms or molecules arrive simultaneously on a heated substrate surface through direct line-of-sight evaporation, resulting in film growth at rates generally much slower than MOCVD. Because of the high vacuum environment and purity of precursors, the resulting films tend to be very pure. Key parameters in the traditional process are the substrate temperature and the ratio of the group V and group III fluxes (V/III ratio). Growth temperatures tend to be somewhat lower than MOCVD, and this provides some advantages in staying below the InN decomposition temperature. Since there is no high-purity solid or liquid source for nitrogen that can be controllably employed in MBE growth of III–V nitride films, the process used is slightly modified to employ a gaseous source of nitrogen and, almost exclusively, to activate that gas using a plasma source. This results in a more complicated flux to the surface involving ionized and/or radical atoms and/or molecules and additional key parameters that influence the relative concentrations of each including pressure, plasma power, and flow rates of gases through the plasma source. Despite these complications, MBE has played a key role in advancing III–V nitride semiconductor research and has resulted in some of the best quality InN films ever produced.^27^ More recently, the enhanced control of the MBE growth process was leveraged to conduct a slight variation on traditional plasma assisted MBE called metal modulated epitaxy (MME) with some advantages for InN and related ternary growth allowing the full range of alloys to be grown for In_1–*x*_Ga_*x*_N.^28,29^

A long-standing method for depositing materials of all types including electronic materials is sputtering.^30^ In this method, either the element of the desired film (indium, for example) or the composition of the film itself (InN, for example) is formed into a solid sputtering target, and then a plasma is used to generate charged species that impinge on the target (often under bias) and eject target atoms/molecules into a medium-to-high vacuum where they are transported to a heated substrate upon which film growth occurs. There are several variations of the process depending on whether the plasma gas is inert (simple sputtering from a target with the desired composition of the film) or not (reactive sputtering from an elemental target or a target with the desired composition of the film) and on the types of plasmas and biasing configurations used (magnetron sputtering being an increasingly popular type). These methods, being well established and involving simpler equipment than MOCVD or MBE, can be more readily available at lower cost. The inherently low temperatures afforded by the method are advantageous for InN and related alloy growth, especially for quaternaries, and the ability to obtain high-purity targets provides improved purity in resulting films. Often sputtered films have a lower degree of crystalline quality,^31^ but this can be improved by exploring the trade-off in temperature and degree of plasma-assist to the growth process (especially for reactive sputtering with nitrogen). More recently, this method has found use in producing or helping to produce electronic-grade III–N films.^32^ Related physical vapor deposition techniques have also been used to grow InN and related alloys including reactive evaporation^33^ and pulsed laser deposition,^34,35^ but these efforts have been limited in number.

Clearly the importance of InN and related alloys is demonstrated by the vast array of methods that have been employed to attempt high-quality film growth. The physical properties of InN-based materials make such growth very challenging. This is particularly true in realizing films with metastable phases (other than wurtzitic), uniform alloy compositions, and alloy compositions within the thermodynamically defined miscibility gaps (except for the metal-modulated MBE methods of recent years). Despite these vast efforts of many decades, there is still no clear method that can provide a manufacturable, scalable process to realize InN-based semiconducting thin films satisfying these challenges, and other methods will need to be developed. We propose plasma-assisted atomic layer growth as a viable process to satisfy these challenges.

## Brief Overview of ALD

**Figure 1:**
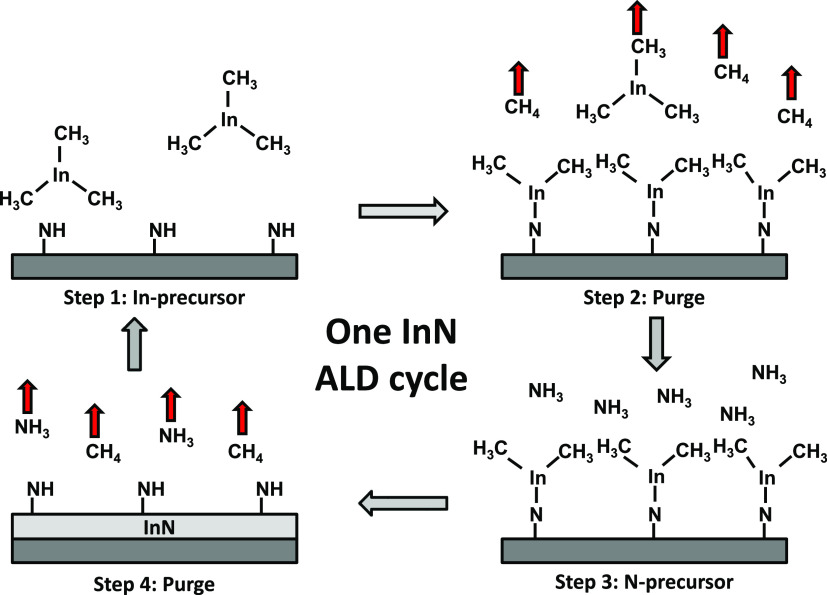
A hypothetical ALD cycle for InN based on TMI (In(CH_3_)_3_) and NH_3_ as In and N precursors, respectively. No such process, with only thermal energy to activate the surface chemistry, has been reported to date; see below for a discussion on why we think that this has not been achieved. The process has, however, been realized by introducing the NH_3_ in an Ar plasma to decompose the NH_3_ molecules prior to reaching the surface.

Atomic layer deposition (ALD) is a time-resolved form of CVD where the central concept is that the precursors for the different elements of the film should never meet in the gas phase. This is typically accomplished by flowing the precursors in alternating pulses into the reactor. The precursor pulses are separated in time by inert gas purges, with the pulsing scheme forming a cyclic process. With no other gases than the inert carrier gas present in the reaction chamber, the only option for the precursor molecules is to react with surface moieties, which they do until all reactive sites within reach are consumed. This means that the surface chemistry is self-limiting and that the film growth is not dependent on the amount of precursor molecules but rather on the amount of surface moieties. A hypothetical ALD cycle for InN is schematically shown in Figure 1, where trimethylindium, In(CH_3_)_3_ (TMI), and ammonia, NH_3_, are used as precursors. It should be noted that, while these precursors are used for CVD of InN, no ALD process has been reported using this chemistry with only thermal energy to activate the surface chemistry. We discuss the possible reasons for this below. The TMI + NH_3_ ALD process for InN has, thus far, only been realized by decomposing the NH_3_ molecules in a plasma prior to reaching the surface.^36^

**Figure 2:**
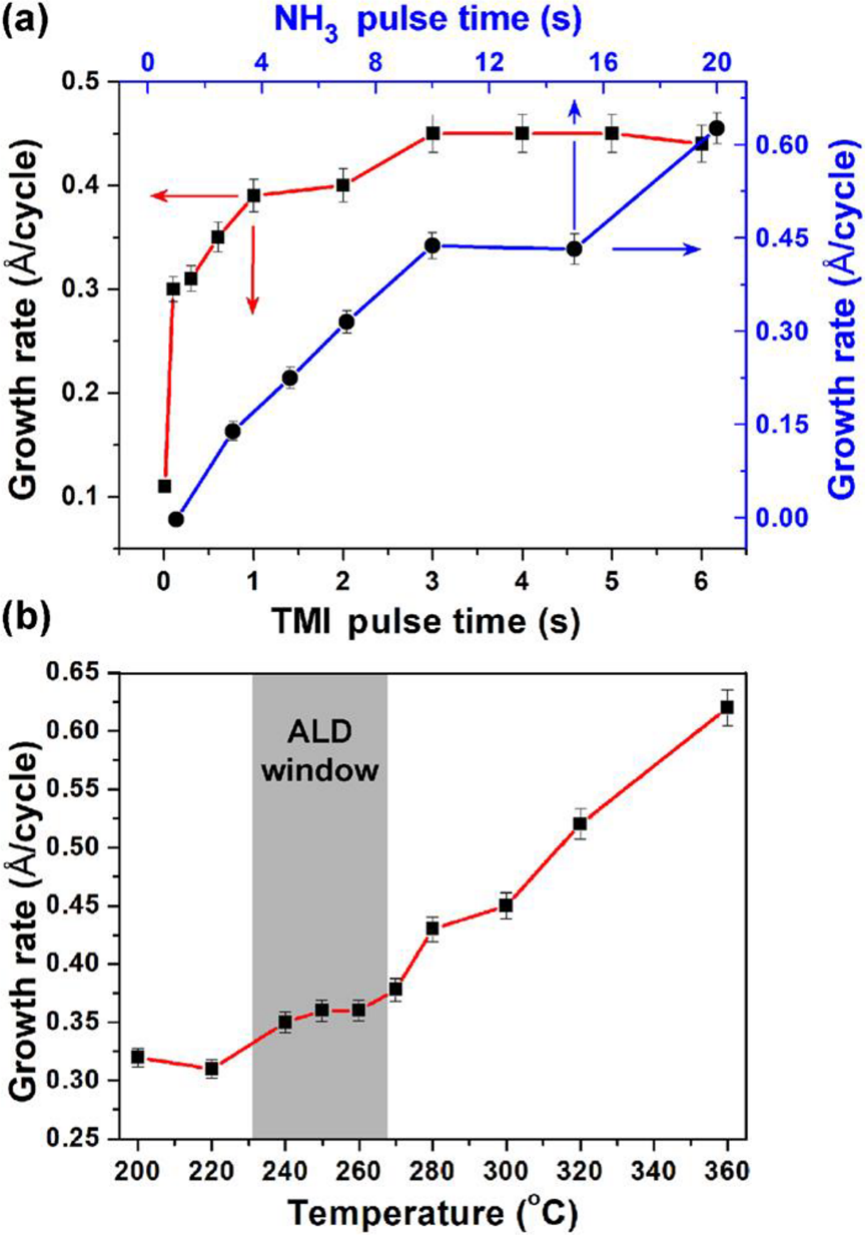
Fundamental characterization of an ALD process is typically done using saturation curves (a), showing a self-limiting surface chemistry for both precursors, here TMI and NH_3_ plasma in an ALD process for InN, and temperature dependence (b), illustrating the stability of the chemisorbed monolayer, again for an InN ALD process using TMI and NH_3_ plasma. Note that an “ALD window” where the GPC is not affected by the temperature is not a sign of self-limiting surface chemistry. Reprinted with permission from Deminskyi et al.^36^ Copyright 2019 American Vacuum Society.

The self-limiting surface chemistry is a central concept of ALD and is demonstrated by a plot with the amount of deposited material versus the amount of supplied precursor, typically given as time for the precursor pulse. The plot (Figure 2a) is commonly referred to as the saturation curve and is used to find the times needed for the precursor pulses to reach the self-limiting behavior of the surface chemistry. Any report of a new ALD process, or a known ALD process used in a different reactor than previously reported, should include saturation curves for both the metal and non-metal precursor pulses. Without presenting saturation curves, the chemistry cannot be claimed to be self-limiting, and thus the process cannot be claimed to be ALD. Since the times for the precursor pulses are major process parameters for ALD, the time for one ALD cycle, i.e., pulse of the metal precursor, purge with inert gas, pulse of the non-metal precursor, and purge with inert gas, typically varies between reactors with different geometries and gas flow patterns. Therefore, it is practical to discuss ALD processes in terms of growth per ALD cycle (commonly denoted GPC) rather than growth rate, which is growth per time unit.

A well-controlled ALD surface chemistry is, of course, dependent on the temperature of the substrate. A temperature that is too low will not provide sufficient energy to activate the surface chemistry and might even lead to condensation of the precursor vapor. A temperature that is too high will instead lead to either desorption of the precursor molecules from the surface or thermal decomposition of the precursor molecules on the surface. Many, but not all, ALD processes display a temperature window where the GPC is constant with temperature, Figure 2b. It should be noted that this so-called “ALD window” only considers the film growth per cycle; other properties of the films such as crystallinity, impurity levels, and roughness, all which can affect the performance of the film in an application, may change in the temperature window.

An extension of ALD is to use a plasma during the non-metal precursor pulse to create more reactive fragments of the precursor. The process is then referred to as plasma enhanced or plasma assisted ALD, PEALD, or PA-ALD, respectively, or simply as plasma ALD. It should be emphasized that the plasma discharge is only activated during the non-metal pulse, generating more reactive species, such as radicals and even ionic species, bearing the non-metal atom. The metal precursor pulse is done in the same way in plasma ALD as in thermal ALD. This points to a general higher reactivity toward surface moieties for the metal precursors than the non-metal precursors. The primary motivation for using plasma ALD is to allow for a lower deposition temperature by replacing some of the thermal energy by electrical energy in the plasma, which is transferred to the surface in the form of metastable species and ions, enabling higher reactivity at lower temperatures. Another motivation is to allow for tuning material properties such as crystallinity^37^ by using a bias to attract the ionic species in the plasma.

The field of ALD has been extensively reviewed several times by several groups. Excellent overviews to highlight are the classical papers by Steven George^38^ and Riikka Puurunen^39^ and the extensive overview of plasma ALD by Harm Knoops et al.^40^ The online ALD resource “Atomic Limits” keeps an overview of all ALD reviews^41^ where the interested reader can find ALD reviews focused on different applications of ALD.

## ALD of InN Using Trimethylindium

A few groups in the world have demonstrated growth of good-quality InN by ALD. Almost all reports have used trimethylindium In(CH_3_)_3_ (TMI) as the In precursor in their work. The first reports of such growth were published in 2013^42^ and made use of a nitrogen plasma generated using an inductively coupled plasma (ICP) source in a mixture of N_2_ and Ar. These first efforts revealed the promise of the ALD method by producing crystalline films on a-plane sapphire and Si(111) substrates, as well as GaN/sapphire templates. The resulting InN films showed that both stable (wurtzitic) and metastable (cubic) phases were possible as a result of the growth process conditions afforded by ALD. This work was followed by others who demonstrated wurtzitic polycrystalline growth on silicon (100) using other plasma sources, e.g., hollow cathode,^43^ and nitrogen sources, e.g., ammonia,^36^ and, most recently, very high structural quality films on more suitable substrates for heteroepitaxy, e.g., (0001) 4H-SiC.^44^ These seminal reports are described in more detail below.

**Figure 3:**
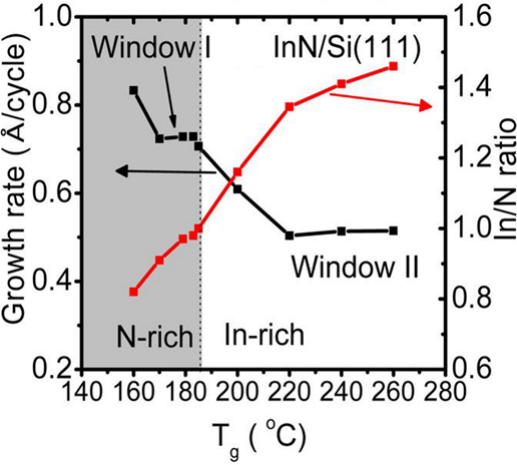
Two ALD windows found for the first reported ALD process using TMI and N_2_ plasma. Reprinted with permission from Nepal et al.^42^ Copyright 2013 American Chemical Society.

In the first ever published efforts to grow InN by ALD, layers were simultaneously grown on a-plane sapphire, semi-insulating Si(111), and MOCVD GaN/sapphire templates.^42^ The GPC vs temperature at optimal growth conditions shows two ALD growth windows (Figure 3). The first is between 170 and 183 °C where it remains constant around 0.73 Å/cycle. The growth rate decreased with increasing temperature from 183 to 220 °C but remained constant again at 0.51 Å/cycle within the second window of *T*_g_ between 220 and 260 °C. The surface morphology of these InN films is smooth and uniform, with root-mean-square (RMS) roughness values calculated from 1 × 1 μm^2^ atomic force microscopy scans to be about 0.45 and 1.17 nm for the films grown at lower and higher temperature growth windows, respectively.

**Figure 4:**
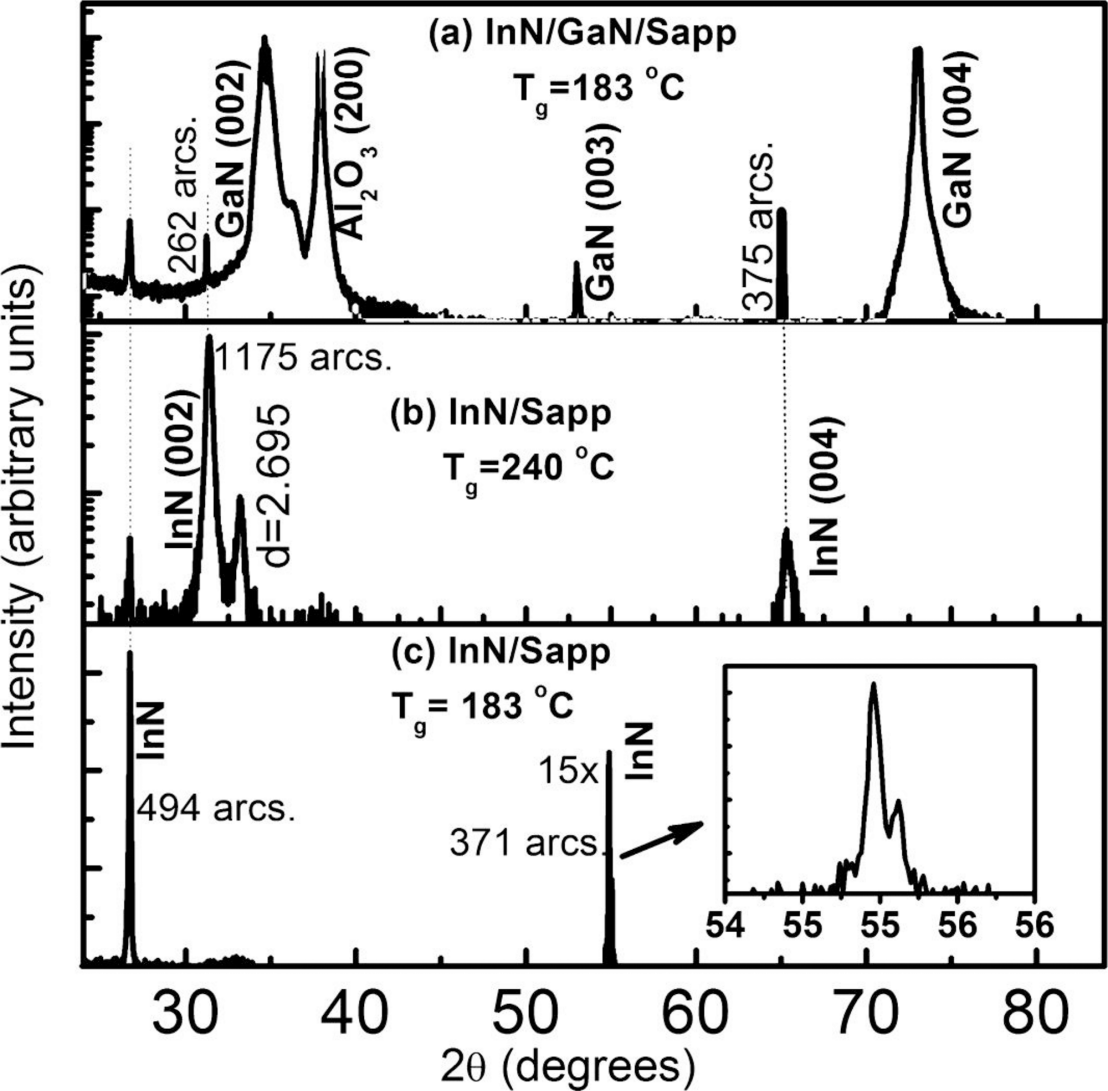
Symmetrical θ/2θ XRD curves of (a) InN/GaN/sapphire grown at 183 °C, (b) InN/a-sapphire grown at ALD growth window II (240 °C), and (c) InN/a-sapphire grown at ALD growth window I (183 °C). InN/a-sapphire at window I is cubic; however, at window II, it has a dominant wurtzite structure. The inset of part c shows a cubic InN second order (222) peak. Both Kα_1_ and Kα_2_ components are resolved indicating the high crystal quality of cubic InN. Reprinted with permission from Nepal et al.^42^ Copyright 2013 American Chemical Society.

X-ray diffraction (XRD) of the InN sample grown on a-sapphire at the low-temperature growth window (Figure 4) shows a distinctly different pattern from that of the most stable wurtizite structure. It does not have the wurtize InN(0002) peak; instead, it has a set of first (26.71°) and second (54.96°) order 2θ peaks that could be indexed to cubic structure. The fwhm values are 494 and 371 arc-secs for the first and second order peaks, respectively, indicating high crystalline quality. The InN sample grown on the a-plane sapphire at the higher temperature growth window formed both cubic and hexagonal phases, with the significantly greater intensity of the (0002) and (0004) peaks compared to the cubic peaks suggesting that the hexagonal phase is the major phase, with a *c* lattice parameter of 5.71 Å. There is a weak cubic InN peak at 26.71° and a hexagonal InN (1011) peak at 33.22°. The fwhm of the (0002) InN peak is about 1175 arc-secs indicating relatively poor crystalline quality, which may arise from the large in-plane lattice mismatches of about 9 and 29% of hexagonal InN with the substrate. However, the lattice mismatch between cubic InN [011] and sapphire [1100] is 2.8%.^42^ The smaller lattice mismatch along the in-plane direction resulted in a high-quality cubic InN film. It is believed that this mismatch provided the necessary strain to stabilize the metastable phase. Based on the g-vector ratio in transmission electron micrographs and the angles between the reflecting planes, the InN layer on a-plane sapphire grown at the low-temperature ALD growth window is determined to be of a FCC NaCl type structure. It was also found that the [100] and [011] cubic InN planes are parallel to [1120] and [0001] planes of the sapphire, respectively.

The NaCl type crystal structure InN grown at the lower temperature growth window (170–185 °C, growth window I) has higher resistivity than the hexagonal phase.^42^ An electron mobility of ∼25 cm^2^/V s was measured for 15 nm thick films, which is similar to the reported value of thick InN grown on sapphire.^45^ However, InN/a-sapphire films grown at the higher temperature growth window (220–260 °C, window II) are highly conducting and have a dominant wurtzite crystal structure and lower electron mobilities.

**Figure 5:**
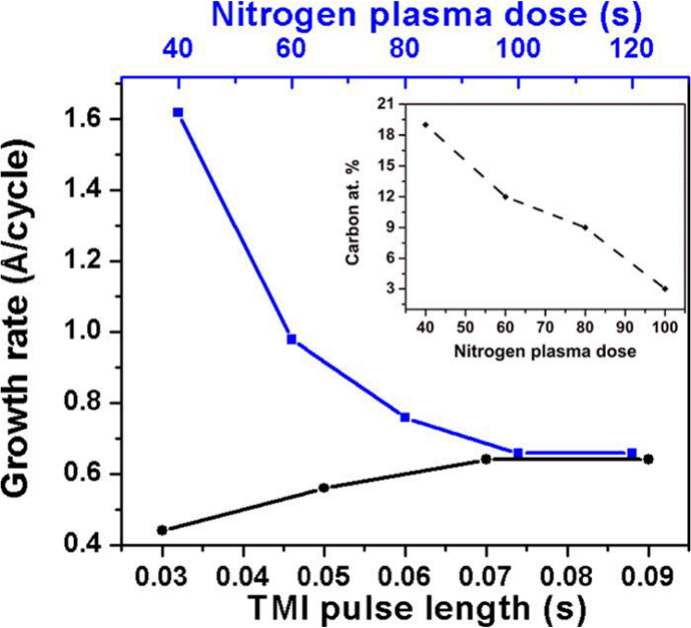
Effect of the precursor dosage on the growth rate at 200 °C. The TMI pulse length was kept constant at 0.7 s for the N_2_ plasma saturation curve, and the N_2_ plasma dose was kept constant at 100 s for the TMI saturation curve. (Inset) N_2_ plasma dose vs carbon at. % present in the bulk of the film. Reprinted from Haider et al.^43^ under a CC-BY license.

Separate efforts were reported subsequent to these initial efforts. In 2016, Haider et al.^43^ reported on self-limiting ALD growth of InN on silicon (100) using a hollow-cathode type plasma source to generate a N_2_ plasma. This study of process parameters identified conditions for self-limited growth from 200 to 350 °C at a rate of 0.5 Å/cycle but required very long plasma times to get low levels of impurities (Figure 5).

The resulting films under these conditions showed wurtzitic polycrystalline structure and were nearly stoichiometric with carbon and oxygen contamination at the film surface that was reduced by an order of magnitude (to 2–3%) in the bulk of the film. Like the work reported above, the films were N rich for lower growth temperatures. The optical bandgap measurements also showed a much larger gap of 1.9 eV than the currently accepted value of ∼0.7 eV. The measured larger bandgap is in line with other reports in the literature where, e.g., In/N stoichiometry and oxygen impurities are expected to affect the bandgap.^46^

**Figure 6:**
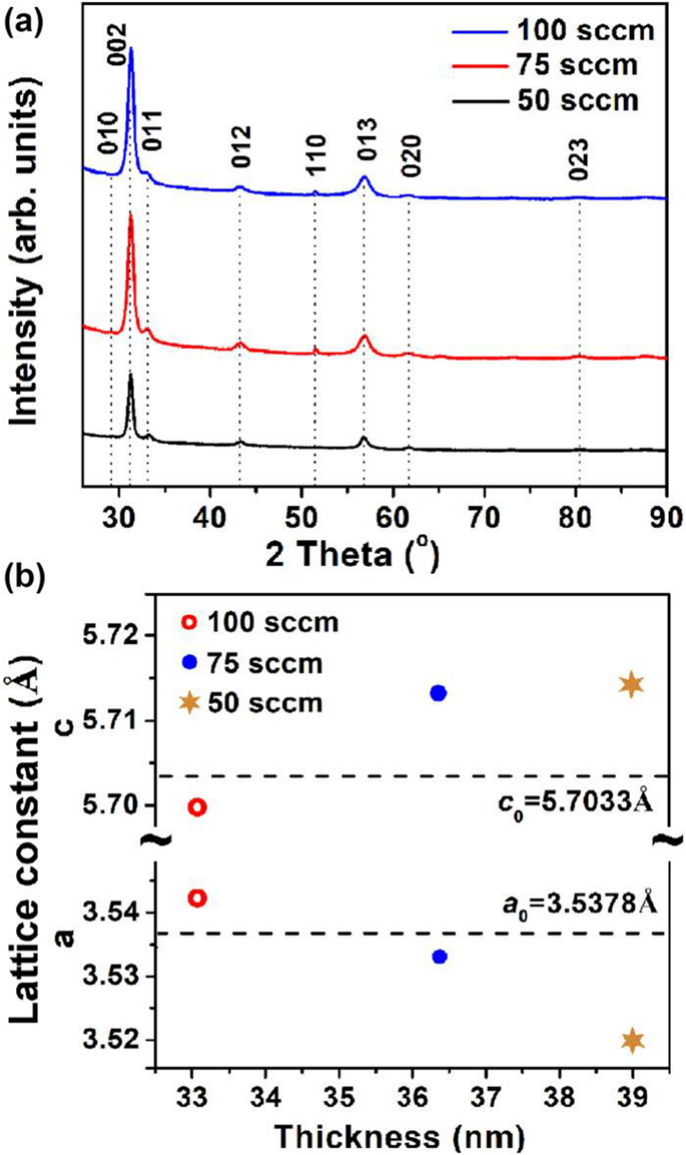
GIXRD pattern (a) and variations of the *a-* and *c-*axis lattice constants (b) of InN film deposited on the Si(100) substrate at 50, 75, and 100 sccm of NH_3_ flow at 320 °C. Reprinted with permission from Deminskyi et al.^36^ Copyright 2019 American Vacuum Society.

More recently, Deminskyi et al.^36^ reported on ALD of InN on Si(100) using ammonia plasmas generated using an inductively coupled microwave source. In this work a very narrow temperature window of 240–260 °C was identified, and the growth rate in this window was 0.36 Å/cycle. The resulting films here were also polycrystalline and single phase (wurtizitic), and the effect of ammonia flow rate into the plasma was a key control in the crystalline quality (Figure 6). Films were nearly stoichiometric and had, in comparison to other ALD reports in InN growth, low levels of carbon (<1 at. %) and oxygen (<5 at. %) in the bulk of the film. The lower carbon contamination is attributed to the more favorable surface chemistry enabled by the NH_3_ plasma as compared to N_2_ plasma. It should be noted that the oxygen impurity level is one of the major problems for InN ALD. MBE and MOCVD are, thanks to lower vacuum levels and higher temperatures, still superior in this aspect.

**Figure 7:**
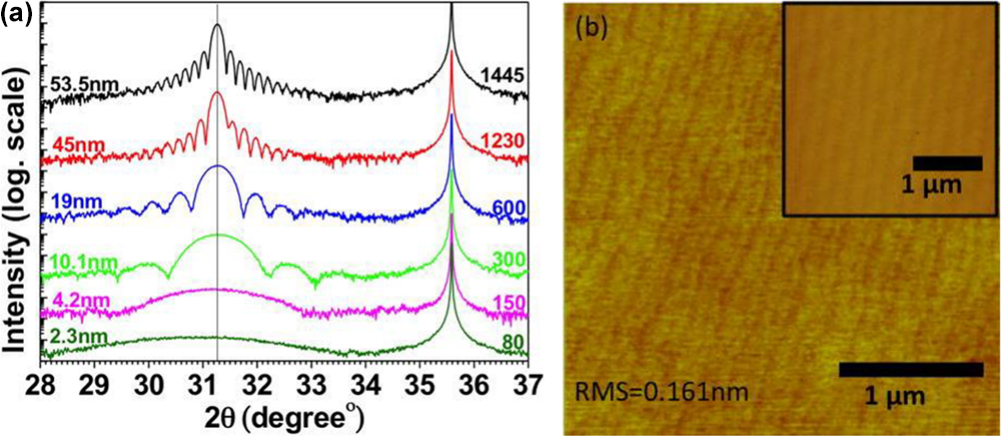
(a) X-ray diffractograms of the 2θ–ω scan of InN films grown with different numbers of ALD cycles on 4H–SiC(0001). The number of ALD cycles and their corresponding film thicknesses determined by fringes are indicated in the plot. The curves are plotted on the log scale and are shifted vertically for visual clarity. (b) An AFM micrograph of a 20 nm InN film grown on the 4H–SiC substrate. Inset: an AFM micrograph of a 4H–SiC substrate prior to the growth of InN. Reprinted from Hsu et al.^44^ under a CC-BY license.

Most recently, Hsu et al.,^44^ from the same group, reported growth of very high structural quality InN using the same ammonia plasma onto on-axis 4H silicon carbide (SiC) substrates for even very thin (<50 nm) films (Figure 7a). In this work, the resulting films (0001) are epitaxially aligned to the underlying SiC(0001) and are very smooth (0.14 nm rms roughness). The film morphology also reproduces the step surface of the substrate, indicating highly conformal coverage (Figure 7b). Transmission electron microscopy studies of these films and their interface with SiC show a very abrupt and low defect interface with strong epitaxial relationships. The quality of these films is highly encouraging for the future of ALD approaches to III–N film growth, especially metastable phases and ternary stoichiometries.

Collectively, these works show the promise of ALD for growing high-quality InN of both stable and metastable structure. They also lay the foundation for the processing space for ALD grown InN which is summarized in Table 1, where three separate groups reported similar ALD growth temperature windows and growth rates using different deposition tools. A general comment on these results is that correct stoichiometry is challenging to achieve.

**Table 1:** Summary of the ALD Processes for InN Growth Using TMI (Trimethylindium)

Group	Substrate	TMI pulse (s)	Plasma	Plasma source	ALD window (°C)	GPC (Å/cycle)	In/N	Ref.
U.S. Naval Research Lab	Si(111)	0.06	N_2_–Ar	RF ICP	220–260	0.51	1.4	(42)
Bilkent University	Si(100)	0.07	N_2_–Ar	Hollow cathode	240–350	0.52	1.1	(43)
Linköping University	Si(100)	3	NH_3_–Ar	RF ICP	240–260	0.36	1.1	(36)

## Working to Understand InN Growth by ALD

Further advancement of the ALD growth of crystalline materials, especially III-Ns, will require a better understanding of the complex growth processes. In recent years, an expanding range of characterization techniques has been adopted for the *in situ* monitoring of ALD processes;^47^ however, the highly contaminating precursors, relatively high pressures, and overall harsh process environments generally preclude various powerful techniques which require ultrahigh vacuum (UHV) unless differential pumping is used. The interested reader is directed to Fukumizu et al.^48^ and Bankras et al.^49^ for examples of unique implementations of *in situ* X-ray photoelectron spectroscopy and reflection high-energy electron diffraction, respectively.

**Figure 8:**
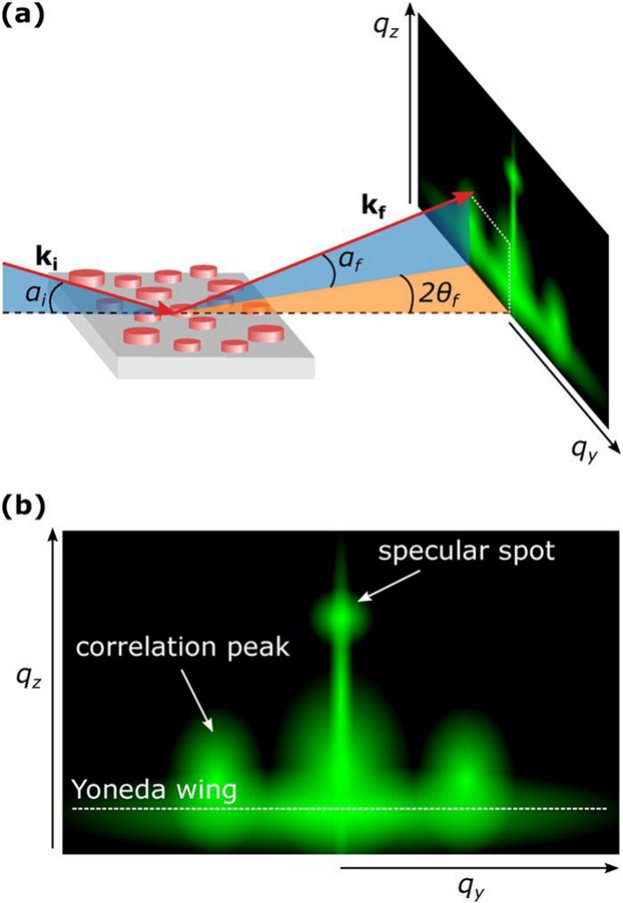
Illustrations of the GISAXS (a) scattering geometry and (b) pattern for island topography. Reprinted with permission from Woodward et al.^62^ Copyright 2022 American Vacuum Society.

An alternative, ALD-compatible approach to *in situ* characterization is to utilize hard X-ray scattering, as the incorporation of X-ray transparent windows on the entry (“upstream”) and exit (“downstream”) sides of the process chamber relative to the incident beam allows for measurement equipment to be placed outside.^50^ Notably, hard X-ray scattering techniques are nondestructive, are readily adapted to any kind of material without suffering from charging effects, and are capable of probing nano- and atomic-scale features at arbitrary pressures and temperatures in real time,^51^ which motivates their use for studies of ALD. Among such techniques, grazing incidence small-angle X-ray scattering (GISAXS) is particularly well suited for the investigation of nucleation and growth kinetics due to its exceptional sensitivity to nanoscale surface topography. In GISAXS, an X-ray beam impinges upon the sample and produces a diffuse scattering pattern that contains information such as the size, shape, and spatial arrangement of surface or buried features. Illustrations of a typical scattering geometry and scattering pattern are shown in Figure 8a and b, in which the scattering intensity is described as a function of in-plane and out-of-plane momentum transfer, *q*_*y*_ and *q*_*z*_, respectively. GISAXS studies of ALD processes have been reported for a broad range of material systems, including various metals,^52−54^ oxides,^55,56^ and nitrides.^57−62^

Researchers at the U.S. Naval Research Laboratory have reported several *in situ* GISAXS studies of InN plasma ALD growth kinetics.^58−62^ The most significant results are summarized and discussed here. The experiments were performed in the G3 hutch of the Cornell High Energy Synchrotron Source using a custom plasma ALD system with an ICP source, X-ray-transparent beryllium windows, and air-cooled dry vacuum pump. This custom system was built to emulate the geometry of a Veeco Fiji system and employed an identical plasma source. More details of the system can be found elsewhere.^63^ The ALD processes used TMI and N_2_/Ar plasma, and the chamber pressure was approximately 0.2 Torr with gas flows. All growths were performed at a growth temperature of 250 °C unless specified otherwise.

**Figure 9:**
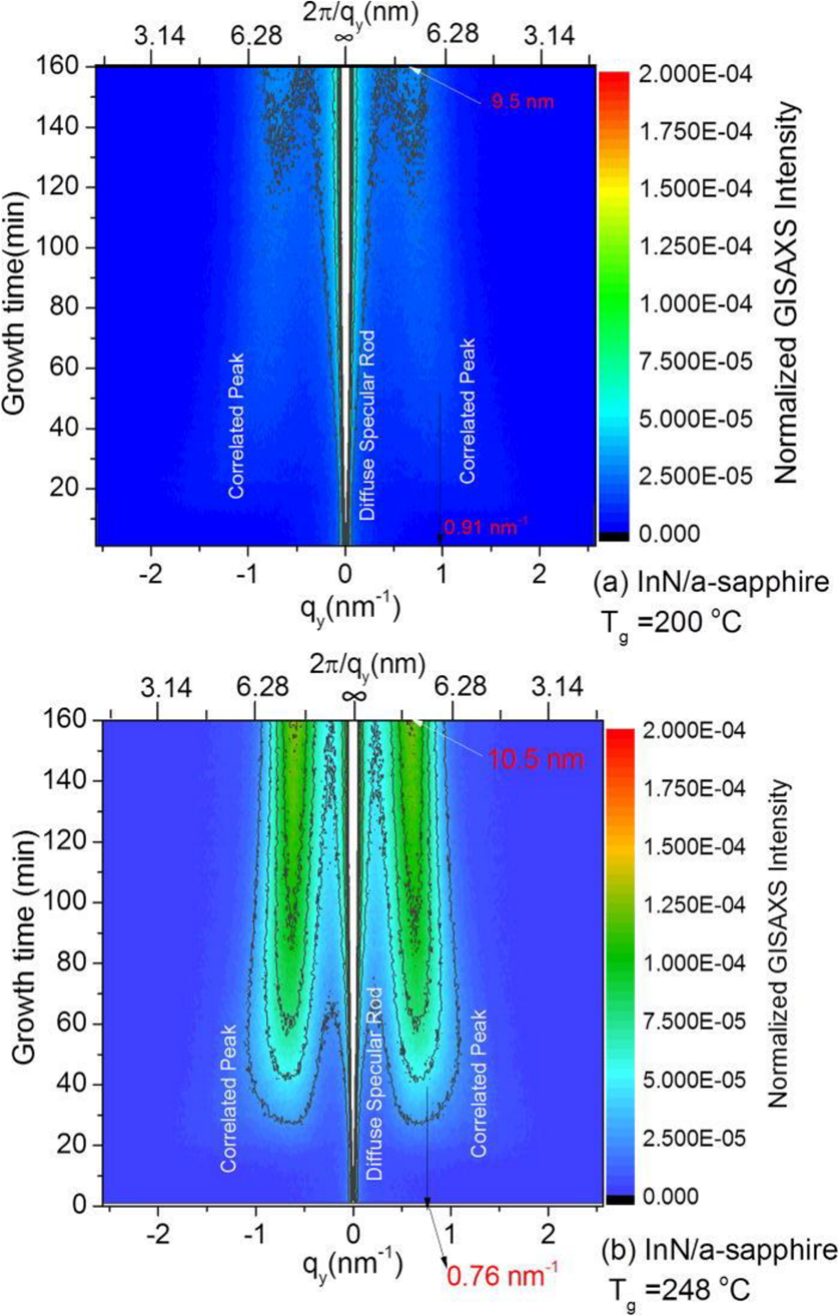
Contour plots of real-time GISAXS intensity evolution of InN/a-sapphire at (a) 200 and (b) 248 °C. Island center-to-center distance *L* is related to the correlation peak position as 2π/*q*_*y*_ (top axis in the plots). The X-ray incidence and exit angles are 0.8° and 0.2°, respectively. Reprinted with permission from Nepal et al.^58^ Copyright 2017 American Vacuum Society.

The first *in situ* GISAXS study of InN PEALD was reported by Nepal et al.,^58^ who investigated the growth of InN on a-plane sapphire at 200 and 248 °C. Distinct island-like topography, evidenced by the appearance of “correlation peaks” in the GISAXS pattern, was observed within 8 cycles or approximately one monolayer of deposition, and persisted throughout the remainder of the growth process, as shown in Figure 9. After nucleation, both samples exhibited a gradual increase in mean island center-to-center distance *L* (inversely related to the *q*_*y*_ position of the correlation peak), which began to saturate after approximately 80 cycles or 10 monolayers, with the saturation being more pronounced for the lower temperature growth process. The initial and final values of *L* were both found to increase with temperature. The higher temperature also promoted an enhancement of the rate of increase in total island volume and in-plane order during the early cycles, which began to saturate at approximately 32 cycles or 4 monolayers and exhibited near-complete saturation after approximately 79 cycles or 10 monolayers. The authors attributed these results to an increase in adatom mobility with temperature and the growth mode becoming more conformal upon complete coverage of the underlying sapphire, thus reproducing the existing InN morphology and preserving the established interisland distance *L*.

Nepal et al. then investigated the influence of plasma exposure duration by utilizing a range of exposure times (*t*_p_) corresponding to undersaturated (15 and 20 s), optimal (25 s), and oversaturated (30 s) conditions.^60^ The GISAXS analysis focused on the evolution of island center-to-center distance *L* and island shape, the latter of which was approximated from a relationship between the scattering intensity decay at high angles and the sharpness of the island sidewalls. While all samples exhibited GISAXS patterns consistent with island topography, *L* was only observed to increase with successive cycles for the plasma ALD processes in which 15 s ≤ *t*_p_ ≤ 25 s. For the oversaturated 30 s plasma condition, *L* remained constant. The most undersaturated plasma condition (*t*_p_ = 15 s) was found to promote bimodal island formation. The optimized and oversaturated plasma exposure conditions exhibited a gradual evolution of the islands from more mounded shapes (e.g., hemispheres) to sharper shapes (e.g., cylinders).

**Figure 10:**
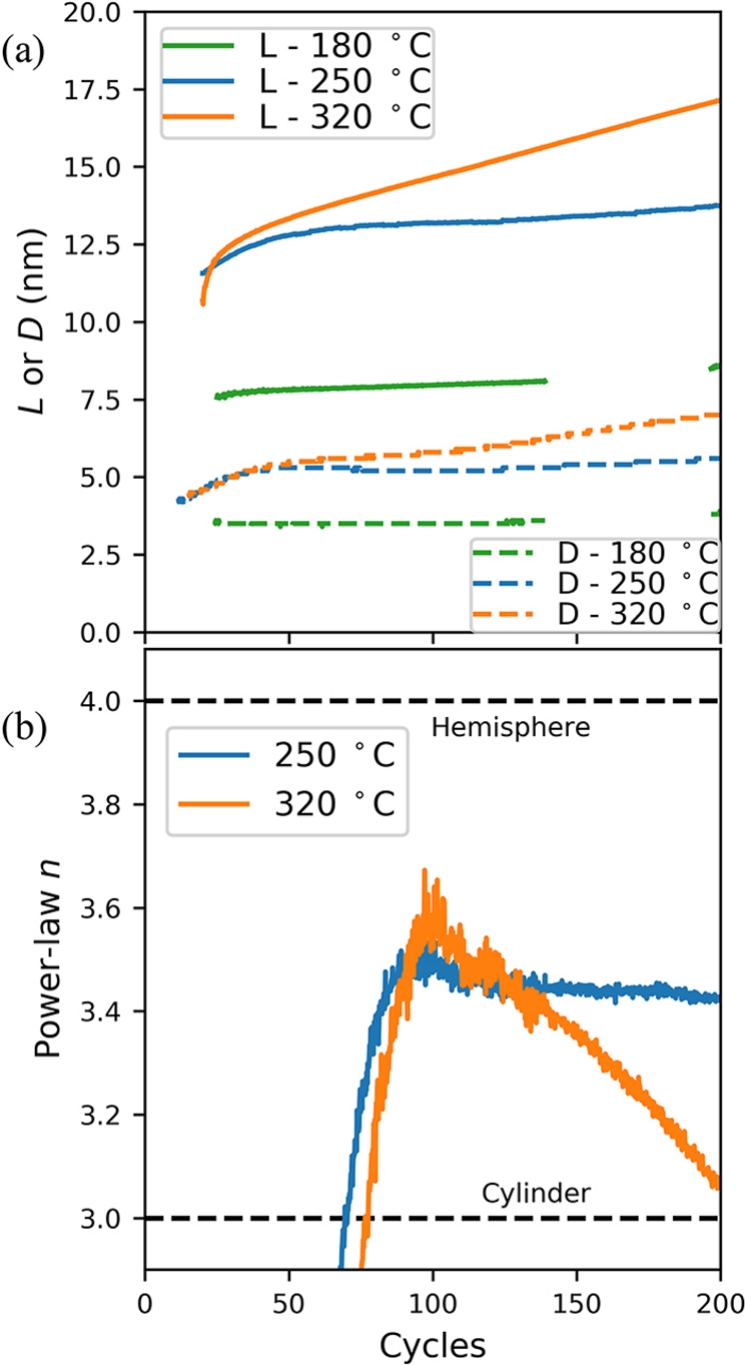
(a) Evolution of mean island spacing *L* determined from correlation peak position and diameter *D* determined by numerical fitting of simulated scattering from cylinders. (b) Evolution of the power-law exponent *n* from *I*(*q*_*y*_) ≈ *q*_*y*_^–*n*^ fit of GISAXS linecuts at high in-plane scattering angle *q*_*y*_, which is related to island sidewall sharpness. Reprinted with permission from Woodward et al.^61^ Copyright 2019 American Vacuum Society.

Woodward et al. investigated plasma ALD of epitaxial InN on freestanding c-plane GaN substrates at 180, 250, and 320 °C.^61^ A known approach to GISAXS analysis based on small-angle scattering theory^64−66^ was utilized to accurately determine the real-time evolution of growth mode and island geometry. Initial island formation was observed after 1.0–2.3 monolayers of two-dimensional growth depending on temperature, from which the InN films were concluded to grow in a strain-driven layer-plus-island (Stranski–Krastanov) mode. As shown in Figure 10a, *L* and mean island diameter *D* were observed to increase with both deposition cycles and temperature, with significantly enhanced island coarsening occurring at the highest temperature of 320 °C, which was attributed to increased adatom diffusion. Increased temperature was also observed to promote the transition of island sidewalls from mounded (e.g., hemisphere) to sharp (e.g., cylinder) shapes, as seen in Figure 10b.

**Figure 11:**
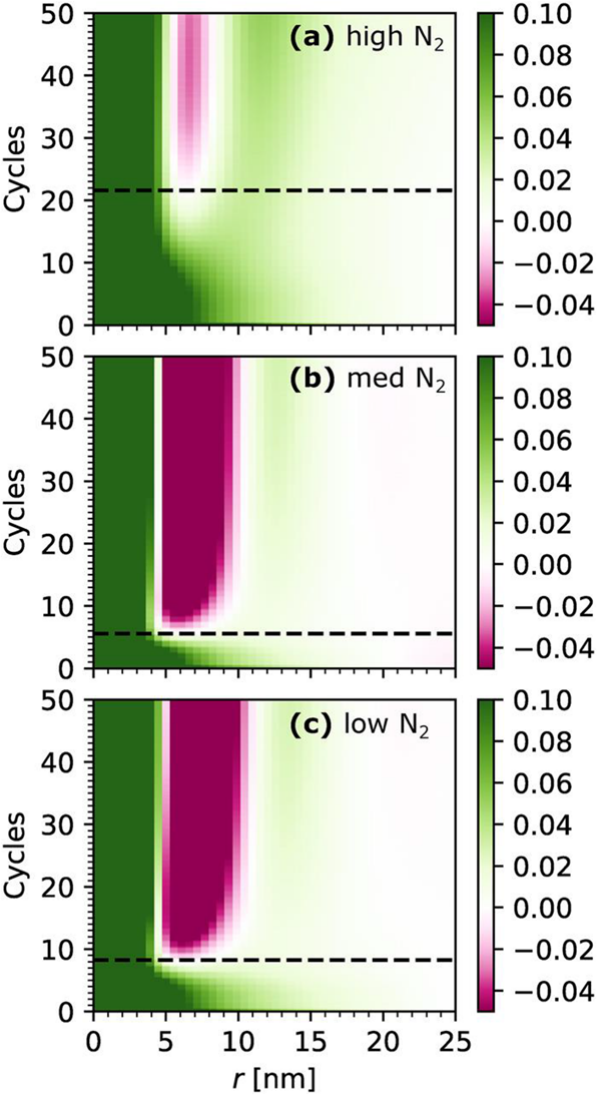
Pseudocolor plots of the Fourier transform of the GISAXS data, in which the appearance of certain features indicates the onset of island growth, indicated by a dashed line: (a) high N_2_, (b) medium N_2_, and (c) low N_2_ plasma regime. Reprinted with permission from Woodward et al.^62^ Copyright 2022 American Vacuum Society.

**Figure 12:**
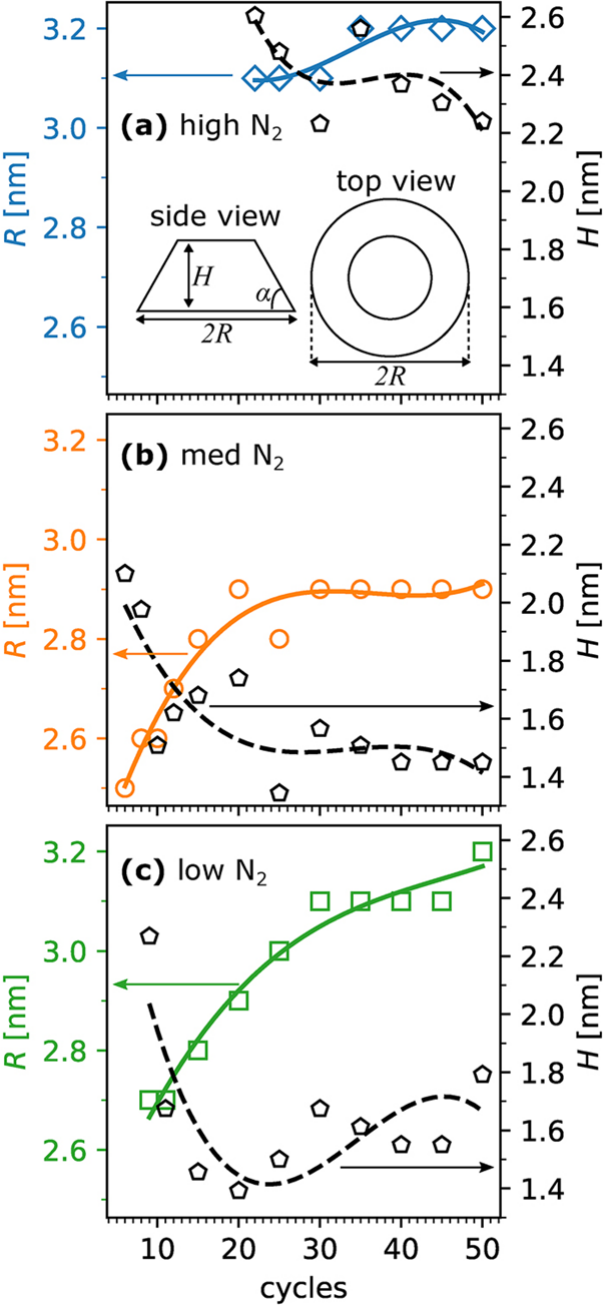
Evolution of the truncated cone base radius *R* and height *H* for (a) high, (b) medium, and (c) low N_2_ plasma regimes. The solid and dashed lines are cubic spline fits of the data points, which act as visual guides. Reprinted with permission from Woodward et al.^62^ Copyright 2022 American Vacuum Society.

Most recently, Woodward et al. investigated the influence of plasma species on the early stage growth kinetics of InN on c-plane GaN,^62^ when the most rapid and dramatic structural changes in the film occur. The concentrations of neutral radical and charged species were determined using optical emission spectroscopy and Langmuir probe measurements.^67−69^ Three different regimes of plasma species generation were explored: low N flux and low ion flux, high N flux and medium ion flux, and high N flux and high ion flux, which were accessed using “high”, “medium”, and “low” N_2_/Ar gas flow fractions, respectively. The transition from two-dimensional to island topography was observed at 2.1, 0.98, and 0.66 monolayers for the high, medium, and low N_2_ plasma conditions, respectively, as shown in Figure 11, from which the growth mode was concluded to depend on the relative density of atomic N radicals, with high concentrations promoting island (Volmer–Weber) growth and low concentrations promoting a layer-plus-island (Stranski–Krastanov) growth mode. The mean island shape was found to be best described as a truncated cone with a 45° incline angle. As shown in Figure 12, the evolution of island size differed dramatically for the cases of Volmer–Weber (medium and low N_2_) and Stranski–Krastanov growth, with the former exhibiting rapid coarsening after nucleation and the latter exhibiting islands which are initially large but undergo only gradual and minor change with continued cycles. Under plasma conditions with a high atomic N density, island coarsening was found to increase with ion flux. Interestingly, the change in topography appeared to occur almost exclusively during the 20 s plasma exposure, with little to no noticeable change occurring during the TMI pulse or purging of parts of the plasma ALD cycle.

To our knowledge, these insights on growth mechanisms could not have been achieved using any other *in situ* technique that has been reported in the literature. The studies presented here have only covered a limited region of the process space, leaving the growth kinetics of many promising approaches to InN plasma ALD, such as processes involving alternative indium precursors (see below), or plasma sources,^43,70,71^ largely uninvestigated. In addition, while the iterative improvements to the equipment, experimental design, and data analysis methods over the course of these studies led to significant increases in both the breadth and depth of information that could be extracted from the experiments, the full capabilities of GISAXS have not been exploited and are constantly increasing. For example, the highly coherent X-ray beams available at specialized beamlines in modern synchrotron light sources can probe the exact rather than the average surface structure, thus revealing information about surface dynamics (i.e., time-correlated fluctuations about the average) that is typically washed out in conventional GISAXS.^72^ Furthermore, there are various other *in situ* X-ray techniques that can be performed simultaneously with GISAXS to provide complementary information about the evolution of, e.g., crystalline structure or deposition volume. Thus, future efforts to investigate InN plasma ALD growth kinetics using GISAXS are highly promising, as they can leverage a highly refined experimental design, new and cutting-edge synchrotron capabilities, and additional dimensions of characterization data which are correlated with the GISAXS data in real time. It is our belief that these future efforts will continue to establish new fundamental understandings of InN plasma ALD which are also broadly relevant to ALD in general and can provide key insights to advance the impact of this growth approach.

## ALD of InN Using Alternative In Precursors

There are very few reports on alternatives to TMI in ALD of InN. In an early report of InN ALD, ethyldimethylindium, InC_2_H_5_(CH_3_)_2_, was used in a spatial ALD reactor with a rotating head supplying the precursors, in a constant flow via two separated outlets, separated in space rather than in time.^73^ This study is also the only study known to us where only thermal energy was used to activate the growth of InN. It is also the only report using this In precursor for ALD of InN; the same group used it also to deposit InGaN in the same reactor setup.^74^

**Figure 13:**
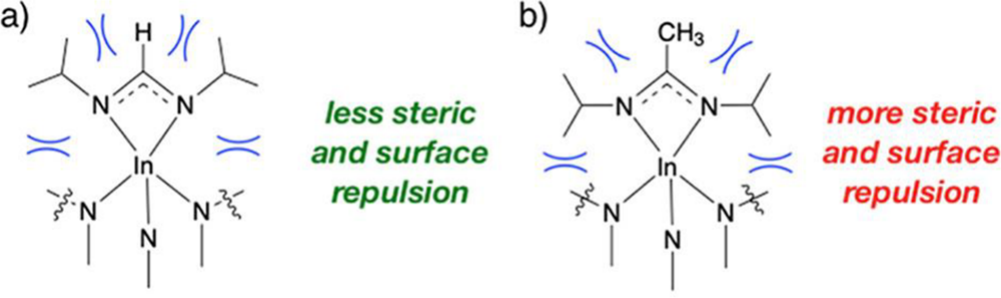
Schematics of the suggested improved surface chemistry for (a) a formamidinate ligand, showing the decrease in steric and surface repulsion of its isopropyl groups in comparison to (b) the amidinate ligand. This model was originally proposed for ALD of In_2_O_3_ from these precursors.^77^ Reprinted with permission from Rouf et al.^78^ Copyright 2019 American Chemical Society.

Cyklopentadienyl indium(I), InCp, is a popular precursor for ALD of In_2_O_3_, but then an oxidizing atmosphere is used capable of oxidizing the In centers from +I to +III in the process. A similarly oxidizing atmosphere is not available to ALD of InN, and InCp can thus not be used in ALD of InN. The field of In_2_O_3_ ALD has developed bidentate ligands forming In–N bonds. Tris(*N*,*N*-dimethyl-*N*′,*N*″-diisopropylguanidinato)indium(III),^75^ tris(*N*,*N*′-diisopropylamidinato)indium(III),^76^ and tris(*N*,*N*′-diisopropylformamidinato)indium(III)^77^ have all been reported in low-temperature ALD processes for In_2_O_3_ with water as an oxygen precursor. These precursors are chemically very similar and differ only in the substituent on the endocyclic carbon atom in the ligand backbone. In a direct comparison between these precursors, when they were tested in ALD of InN with a NH_3_ plasma as a nitrogen precursor, it was seen that the quality of the InN films improved with a smaller substituent on the endocyclic carbon.^78^ The formamidinate ligand with the smallest substituent, a hydrogen atom, rendered films with lower impurity levels and higher crystalline quality of the InN films than the amidinate ligand, where the substituent is a methyl group. The guanidainate ligand, with a dimethylamine group as a substituent, rendered the lowest quality films and was also found to be a poor precursor, prone to decompose upon sublimation into the ALD reactor.^79^ In the first report on the indium formamidinate precursor,^77^ it was speculated that the favorable ALD chemistry seen in ALD of In_2_O_3_ was due to the small substituent, allowing the isopropyl groups on the ligand nitrogen atoms to bend up from the surface to reduce the steric repulsion with the surface (Figure 13). This hypothesis was supported by computational chemistry studies of the surface chemistry of the three precursors, i.e., guanidinate, amindinate, and formamidiante, on the InN surface.^78^

**Figure 14:**
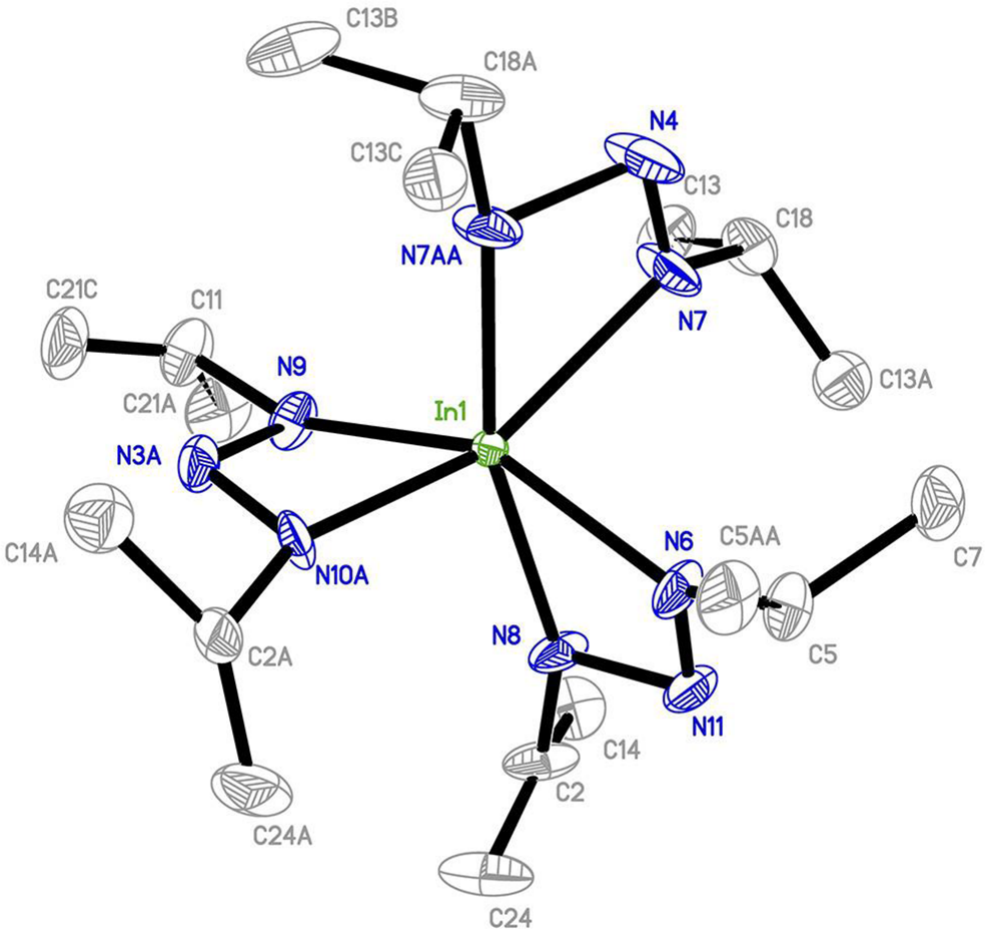
X-ray crystal structure of the tris(1,3-diisopropyl-triazenide)indium(III) precursor with thermal ellipsoids at the 50% probability level. All hydrogen atoms were removed for clarity. Reprinted from O’Brien et al.^80^ under a CC-BY license.

The trend discovered with the size of the substituents was taken further by the triazenide ligand, where the endocylic carbon atom was replaced with a nitrogen atom. Since nitrogen typically makes three bonds instead of four bonds for the carbon atom, the substituent on the endocyclic position could be omitted. The tris(1,3-diisopropyl-triazenide)indium(III) precursor (Figure 14) was synthesized and found to be an excellent precursor for InN ALD, affording epitaxial films with very low impurity levels.^80^

## ALD of In_1–*x*_Ga*_x_*N and In_1–*x*_Al*_x_*N

Following the success of InN by ALD, In_1–*x*_Ga_*x*_N and In_1–*x*_Al_*x*_N have also been grown by ALD. Practically, two different metals can be introduced individually or simultaneously in the ALD growth process.^81^ Since the metals and N must be separated, the process that introduces one metal at a time can be generalized as a supercycle approach, while the one that introduces both metals at time is typically called co-dosing.

A supercycle approach can be seen as a combination of two binary ALD processes. Thus, the growth of In_1–*x*_Ga_*x*_N is done by a repeating cycle (*k*) that is comprised of a number (*m*) of InN cycles (*U*_InN_) and a number (*n*) of GaN cycles (*U*_GaN_). A complete supercycle ALD of In_1–*x*_Ga_*x*_N can be formulated as (*m*·*U*_InN_ + *n*·*U*_GaN_) × *k*. This method has been used to grow both In_1–*x*_Ga_*x*_N^82,83^ and In_1–*x*_Al_*x*_N^84^ with the In content tuned by varying the ratio between *m* and *n*. However, the eventual In content is not only correlated with *m* and *n* but also influenced by the GPC of InN and GaN. According to the rule of mixture,^85^ the thickness and In content of an In_1–*x*_Ga_*x*_N film grown by supercycle ALD can be determined by the following equations:
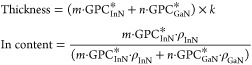
GPC_InN_^*^ and GPC_GaN_^*^ are the GPC of InN and GaN in the growth of In_1–*x*_Ga_*x*_N, and *ρ*_InN_ and *ρ*_GaN_ are the atomic densities of InN and GaN. Ultimately, the supercycle ALD approach allows the formation of a digital alloy. A digital alloy is essentially a short-period superlattice in which the period is based on monolayers. A repetition of altering 1 InN monolayers and 1 GaN monolayers stacking on top of each other has physical properties as homogeneous In_0.5_Ga_0.5_N. By varying the number of monolayers for InN and GaN in each period, the eventual energy gap can be tuned, opening a new route to optoelectronics applications based on such sophisticated materials. Despite the fact that ALD is often seen as “layer-by-layer” growth, achieving a condition that the film is grown as a stacking of altering InN and GaN monolayers is challenging. Frequent switching between two different processes causes significant deviation on GPC so that the GPC_InN_^*^ and GPC_GaN_^*^ are different compared to the GPC_InN_ and GPC_GaN_ determined from the binary InN and GaN ALD processes. Consequently, the thickness and compositions are deviated from theoretical prediction.^82^ According to our recent study,^83^ the GPC of InN decreases more dramatically than that of GaN in a supercycle ALD process for an In_1–*x*_Ga_*x*_N film. However, the In content was found to be higher than predicted from the above model, despite the significant reduction of GPC_InN_ which should also lead to the reduction of In content. The mechanism of this controversial behavior is not yet understood and requires further investigation.

An alternative deposition route to In_1–*x*_Ga_*x*_N is to mix solid precursors and co-sublime them from one sublimator into the ALD reactor. This has been demonstrated using the indium triazenide, discussed above,^80^ and the gallium analogue with the same ligand.^86^ It was shown that, since these two precursors have very similar sublimation temperatures and ALD behaviors, they can be mixed as powders and co-sublimed into the ALD reactor.^87^ The value of *x* in the resulting In_1–*x*_Ga_*x*_N film can be controlled by the mixing ratio in the sublimator, the sublimation temperature, and, to some extent, the deposition temperature. Using this approach, it was possible to deposit a near In_0.5_Ga_0.5_N film with a band gap of 1.94 eV. The film grew epitaxially directly on 4H-SiC(0001) substrates, and cross-sectional TEM and EDX mapping showed no evidence of phase separation. It was, however, noted that the film had a composition gradient and was more Ga rich toward the SiC substrate, with the composition In_0.18_Ga_0.82_N, and more In rich at the top, with the composition In_0.82_Ga_0.18_N. This was speculated to be caused by the lattice mismatch to the SiC substrate.

## The Problem of Using a Thermal ALD Process

All reports above, except for the one early report where InN is deposited in a spatial ALD reactor from In(CH_3_)_2_C_2_H_5_,^73^ are done with plasma activation of the nitrogen source. The thermal process using In(CH_3_)_2_C_2_H_5_ has never been repeated. All other processes using TMI or In precursors with bidentate ligands have used plasma activation of the N precursor. It thus seems that ALD of InN requires the more reactive N species that can be created in a plasma discharge. ALD of InN used plasmas with N_2_ or NH_3_ as the feed gases. Argon is typically used to facilitate the plasma ignition, but it is not reactive in the film deposition. In a thermal process using NH_3_ as the N precursor, the slow decomposition kinetics of ammonia means that the substrate will only be exposed to NH_3_ molecules in the time frame and temperature of a typical ALD process,^88^ i.e., a few seconds and a few hundred degrees Celsius. By optical emission spectroscopy it has been shown that plasma discharges for ALD of InN in N_2_–Ar mixtures contain atomic nitrogen, N_2_(*A*^3^ ∑_*u*_^+^), and N_2_^+^,^57,62^ and NH_3_–Ar plasmas contain NH, N_2_^+^, and atomic hydrogen.^36^ These plasma species are more reactive in ALD than NH_3_, and it is therefore expected that the surface reactions between the nitrogen species and the In-terminated surface are more spontaneous in plasma ALD, compared to thermal ALD.

Since crystalline AlN and GaN have been deposited in thermal ALD processes from TMA^89^ (Al(CH_3_)_3_) and TMG^90^ (Ga(CH_3_)_3_), respectively, it is noteworthy that no reports on thermal ALD of InN from TMI are found in the literature. Given that the TMI pulse is the same, i.e., no plasma is on, in both thermal and plasma ALD, and since plasma ALD of InN from TMI has been reported by several groups, chemisorption of TMI onto an InN surface terminated with some type of −NH_*x*_ moieties should be favorable. The problematic step for a thermal InN ALD process should therefore be in the surface chemistry during exposure to NH_3_. By using computational chemistry to compare the ligand exchange reaction when a NH_3_ molecule replaces a methyl group on a methyl terminated InN(0001) surface to the same reaction on a methyl terminated GaN(0001) surface, it has been shown that the ligand exchange has a much higher energy barrier on the InN surface.^91^ This will make the process much less likely to happen on the InN surface, compared to the GaN surface, meaning a lower probability for a thermal InN ALD process with NH_3_ as the N precursor. The thermodynamic stability of NH_*x*_ surface groups is also much lower on InN, compared to on GaN.^92^ From strict thermodynamics, it is more favorable for surface NH_*x*_ to desorb as NH_3_ than remain on the InN(0001) surface. However, our attempts to instead use hydrazine, N_2_H_4_, together with TMI in a thermal ALD process in our lab at Linköping University have not been fruitful, nor have our experiments to use the indium triazenide together with thermal NH_3_.

**Figure 15:**
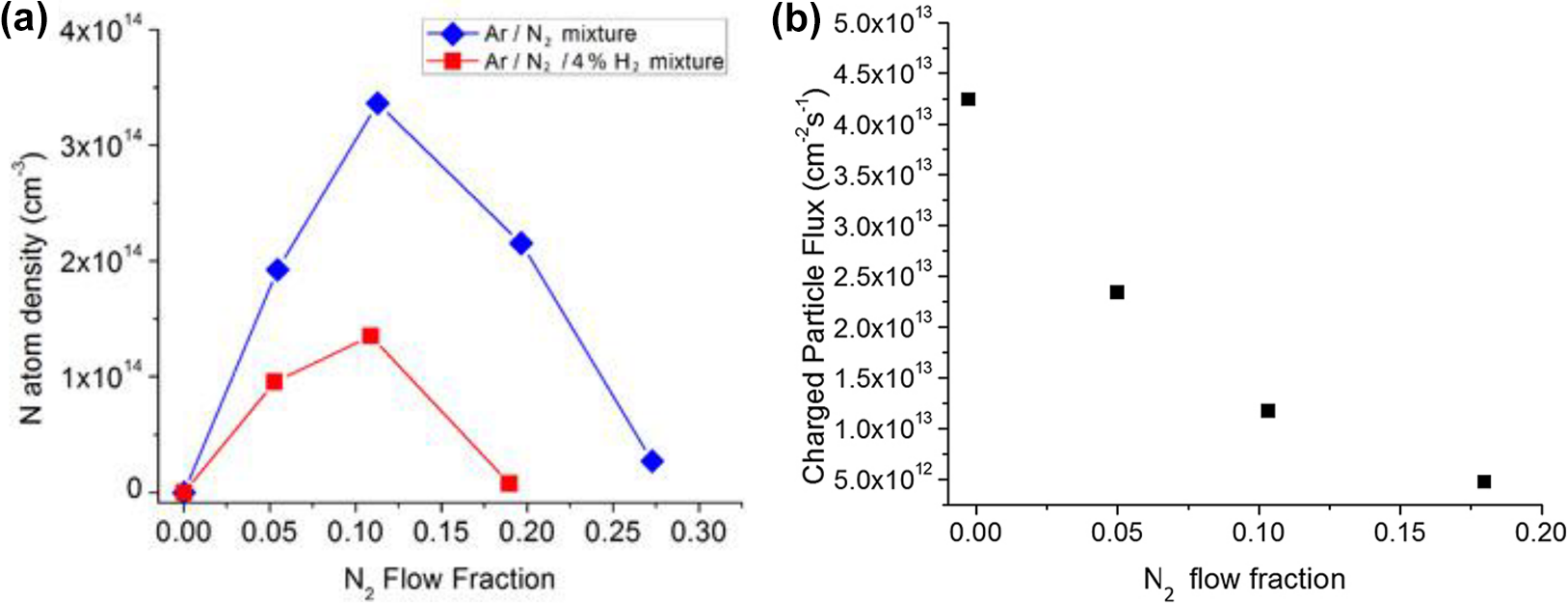
(a) N atom densities as a function of the N_2_ flow fraction into the ICP operated at 300 W with and without the presence of a trace admixture of H_2_. The total flow was 275 sccm. The total neutral pressure for these cases was 340 ± 10 mTorr. (b) Positive ion flux at the grounded surface as a function of N_2_ flow fraction for 300 W of plasma power at a neutral pressure of 300 mTorr. H_2_ was not present for these measurements. Reprinted with permission from Boris et al.^59^ Copyright 2018 American Vacuum Society.

It thus seems that the plasma has a significant role to play in ALD of InN, albeit one which is challenged by the complex relationships between the process parameters (e.g., gas flows, pressure, power) and the plasma species generation and between the plasma species generation and the resultant film properties. The role of the plasma in plasma ALD can be summarized as the delivery of reactive species and energy to the growth surface, driving both surface chemistry and kinetics.^93^ Plasma diagnostic studies of the species generation in an ALD system with ICP source have found that the N atom density in an N_2_–Ar plasma peaks at an N_2_ flow fraction of approximately 0.10 and drops off rapidly as the flow fraction is either increased or decreased, as shown in Figure 15.^59^ Meanwhile, Langmuir probe measurements in the vicinity of the substrate position showed a monotonic increase in positive ion flux as the N_2_ flow fraction decreased, with the 0.05 flow fraction exhibiting a factor of 5 increase compared to the 0.20 flow fraction (see Figure 15b). The reactive neutral flux was estimated to be several orders of magnitude greater than the ion flux, suggesting that it is the former which dominates the surface interactions; however, the energy flux delivered by the ions to the topmost ∼1 nm surface depth was noted to be potentially sufficient to drive nonequilibrium chemistry.

**Figure 16:**
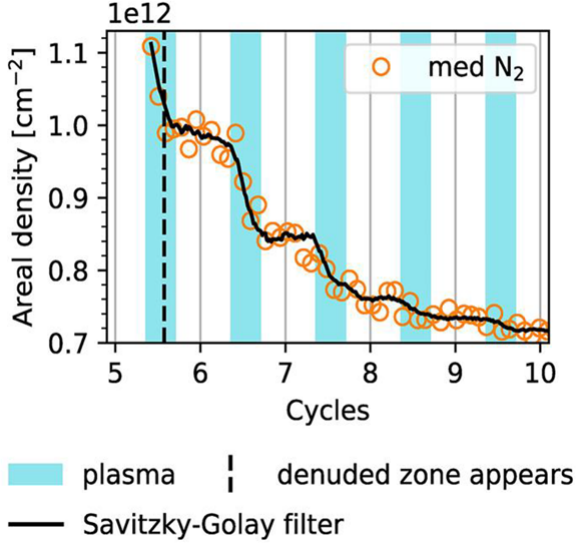
Evolution of island areal density for InN grown on c-plane GaN using N_2_–Ar inductively coupled plasma, monitored by *in situ* GISAXS. The solid black line shows the result of applying a Savitzky–Golay filter to the data points, and the dashed line indicates the initial appearance of the denuded zone. For clarity, every fifth data point is shown by a marker. Reprinted with permission from Woodward et al.^62^ Copyright 2022 American Vacuum Society.

As discussed previously, both atomic N and ionic species have been found to exert a significant influence over the InN plasma ALD growth kinetics, including the growth mode and coarsening behavior. Notably, the growth kinetics have been observed to be dominated by the plasma exposure part of the ALD cycle,^62^ as shown in Figure 16. Such results highlight the importance of both reactive and energy-providing plasma species in controlling InN plasma ALD growth processes and show the potential for deliberately accessing different plasma regimes to tune the kinetics.

## Outlook

This work has demonstrated the potential for ALD of InN and related alloys, but much work remains to be done including better understanding of the role of plasma in such processes, more extensive electrical characterization of carriers in resulting films, improved control of impurities—especially carbon and oxygen—and exploration of combinations of plasma ALD with conventional semiconductor growth methods such as MOCVD and MBE, briefly reviewed here, to enable novel and advanced device structures.

The exact reason ALD has an advantage over continuous CVD processes is still not clear. In continuous CVD processes, deposition of InN requires higher temperatures than in ALD,^94^ since ALD depends, at least partially, on the stability of the chemisorbed monolayer. A key difference is that all ALD processes used plasma to activate the nitrogen chemistry. Plasma activation of the nitrogen chemistry has been found to be highly beneficial also for continuous CVD of InN.^95^ It has been suggested that growth of InN by continuous CVD depends to a large degree on gas-phase reactions initiated from Lewis adducts formed between TMI and NH_3_.^96^ Interestingly, pulsed flow of TMI into a continuous flow of NH_3_^97,98^ and pulses of TMI and NH_3_ with slight overlap^99^ have been reported to deposit good quality continuous InN films with thickness exceeding 75 nm. Time-resolving the supply of TMI and NH_3_ will change the gas-phase chemistry and should reduce the level of formation of adducts.

These studies concluded that separating TMI and NH_3_ was advantageous for InN deposition, pointing to the importance of the dynamics in the precursor supply for growth of InN. We have also shown that the purge time in ALD is an important parameter that modulates the reaction mechanism and its dynamics and strongly influences the deposited InN quality. In attempts to mimic a more continuous CVD process in our ALD reactor, we decreased the time for the purges between the TMI pulse and the NH_3_ plasma, from 8 to 0.1 s. This resulted in deteriorated film morphology and decreased GPC. This was attributed to insufficient time for the gas exchange in the ALD reactor, allowing TMI and NH_3_ species to meet in the gas phase.^94^ Further studies are needed to better understand why ALD technology is such an enabler for InN and its alloys.

The ALD processes for InN require a plasma to deposit film; no thermal processes have been demonstrated yet. The exact role of the plasma is another area that needs to be understood to advance InN ALD. Recent efforts highlighted here have provided initial insights into the role that plasma-generated species play in the plasma ALD^100,93,62,101^ including the importance of controlling charged and radical species fluxes independently. These insights suggest that it may be important to develop novel plasma sources that provide such control in order to take full advantage of the excited species that enable lower temperature growth and the metastable alloys that can result.

In general, InN films have been difficult to characterize, as a result of their surface defectivity and potential to form a conducting surface layer. This challenge is further complicated when films or component layers of digital alloys are ultrathin, as is the case with ALD films. Certainly, dedicated efforts with perhaps more sophisticated characterization methods to accurately measure the electrical transport properties will be needed.^2,102^ The surface morphology of ALD grown InN is an area for improvement. Top-view images of the deposited films are scarce in the literature, but the examples available suggest that the films are not of the mirrorlike smooth morphology that the semiconductor industry has come to expect.

Another challenge exacerbated by plasma ALD is contamination with carbon and oxygen. The carbon from metal–organic precursors can be challenging to convert to sufficiently volatile byproducts at the low temperatures used for growth. This has been largely overcome by using precursors with In–N bonds rather than In–C bonds.^78,80^ However, the level of carbon impurities in these films has been measured by X-ray photoelectron spectroscopy (XPS), with a detection limit of about one atomic percent. Acceptable levels for unintentional impurities in a semiconductor material are typically 10^14^–10^15^ cm^–3^, i.e., much below the XPS detection limit. Much work remains before ALD can be said to produce carbon free InN.

This is also true for oxygen impurities. InN is a very oxyphilic material, and the equipment and processing conditions used for plasma ALD, where oxygen contamination from poor vacuum conditions is a constant concern, need to be addressed.^103^ One course ripe for investigation is how plasma species might be used to reduce such contamination through a combination of radical/ion balance control and small additions to the input gas mixture to the plasma. Such advanced insights could be employed in more complex atomic layer growth cycles that incorporate a “cleaning” step in the cycle.

A key opportunity enabled by the advances highlighted in this review is to combine the metastable materials made possible by plasma ALD with conventional material growth methods, e.g., MOCVD or MBE, to realize novel device structures simply not possible by conventional growth methods alone. By integrating ALD tools with MBE or MOCVD tools, one can potentially create novel heterostructures with electrically pristine interfaces for a range of electronic (high-power, high-frequency transistors) and optoelectronic (emitters and detectors in the visible to near-infrared) applications. Further, these nonequilibrium growth processes could play an important role in advancing the family of “new nitrides” highlighted in a recent paper^104^ to include epitaxially integrated superconductor (niobium nitride), ferroelectric (scandium aluminum nitride), piezomagnetic (gallium manganese nitride), and ferrimagnetic (manganese nitride) materials.

Finally, and as described above, ternary nitrides based on InN have been deposited by ALD. This subfield is more virgin and needs to be better understood in terms of its limits to the compositions possible to deposit. Quaternary materials based on InN, e.g., In_1–*x*–*y*_Al_*x*_Ga_*y*_N, could perhaps also be realized by ALD, further widening the possibilities of the III–N technology.
